# Human iPSC-Derived Vascular Smooth Muscle Cells in a Fibronectin Functionalized Collagen Hydrogel Augment Endothelial Cell Morphogenesis

**DOI:** 10.3390/bioengineering8120223

**Published:** 2021-12-18

**Authors:** Kaiti Duan, Biraja C. Dash, Daniel C. Sasson, Sara Islam, Jackson Parker, Henry C. Hsia

**Affiliations:** 1 Section of Plastic Surgery, Department of Surgery Yale School of Medicine, Yale University, New Haven, CT 06510, USA; kaiti.duan@yale.edu (K.D.); daniel.sasson@yale.edu (D.C.S.); sara.islam@yale.edu (S.I.); jackson.parker@yale.edu (J.P.); 2 Department of Biomedical Engineering, Yale University, New Haven, CT 06511, USA

**Keywords:** induced pluripotent stem cell, vascular smooth muscle cells, fibronectin, vascular network formation, angiogenesis, VEGF, bFGF

## Abstract

Tissue-engineered constructs have immense potential as autologous grafts for wound healing. Despite the rapid advancement in fabrication technology, the major limitation is controlling angiogenesis within these constructs to form a vascular network. Here, we aimed to develop a 3D hydrogel that can regulate angiogenesis. We tested the effect of fibronectin and vascular smooth muscle cells derived from human induced pluripotent stem cells (hiPSC-VSMC) on the morphogenesis of endothelial cells. The results demonstrate that fibronectin increases the number of EC networks. However, hiPSC-VSMC in the hydrogel further substantiated the number and size of EC networks by vascular endothelial growth factor and basic fibroblast growth factor secretion. A mechanistic study shows that blocking αvβ3 integrin signaling between hiPSC-VSMC and fibronectin impacts the EC network formation via reduced cell viability and proangiogenic growth factor secretion. Collectively, this study set forth initial design criteria in developing an improved pre-vascularized construct.

## 1. Introduction

Wound healing is a complex biological process and requires the coordination of several molecular events such as homeostasis, inflammation, cell proliferation, angiogenesis, and matrix remodeling. Chronic wounds fail to follow these ordered wound healing processes and result in wounds that do not heal and ultimately require extra care [1]. Tissue-engineered constructs provide an alternative to autologous grafts for the treatment of these non-healing wounds [1,2]. These constructs are bioengineered scaffolds that contain ECM or a biopolymer of choice and/or antibiotics, growth factors, and human cells [3,4,5,6,7]. However, the constructs fail to stably engraft in chronic wounds with vascular insufficiency requiring additional interventions [8]. The primary reason for this failure is the lack of vascularization in the implanted tissue-engineered constructs and cell death [9]. A pre-vascularized construct improves engraftment and survival after transplantation and helps in establishing a functional vasculature in the host [10]. In sum, this evidence suggests that pre-vascularized hydrogels can promote chronic wound healing.

Morphogenesis of endothelial cells (ECs) is a prerequisite for vessel formation [11]. During morphogenesis, the ECs proliferate, migrate and self-assemble to form a tube-like structure [12]. Studies reveal that ECM such as fibronectin regulates vascular remodeling via integrin signaling. Gerecht and colleagues in one such study demonstrated the role of fibronectin in matrix assembly and EC morphogenesis. They studied the role of polymerized fibronectin on vascular tubulogenesis on a decellularized matrix. Most importantly, they showed the importance of fibronectin and integrin αvβ3 and α5β1 in EC morphogenesis [13].

One of the vascular tissue engineering strategies is to utilize stromal cells to rebuild patent vessels in a hydrogel matrix for subsequent transplantation [14]. The generation of vascular networks containing vascular smooth muscle cells (VSMC) is highly relevant as they play an integral part in forming vasculatures both in a natural and pathological process [15]. In more recent works, we and others have demonstrated the derivation of VSMC from human-induced pluripotent stem cells (hiPSC) [16,17].

Human iPSCs have been a vital part of tissue engineering for their promises in disease modeling and potentials for regenerative medicine [18,19,20]. In addition, the manufacturing methodologies of hiPSC do not raise the ethical concerns that human embryonic stem cells do, thus making hiPSC attractive candidates in basic science research. In particular, the establishment of an efficient and high output hiPSC derived VSMC (hiPSC-VSMC) generation process has allowed researchers to study vascular diseases and proangiogenic behaviors [17,21,22]. We have established a protocol to generate a pure population of hiPSC-VSMC without the need for additional purification steps [21,22]. Our recent studies on the hiPSC-VSMC paracrine secretion profile have shown that hiPSC-VSMC, under the influence of the microenvironment, physically or biochemically, secretes numerous proangiogenic growth factors and anti-inflammatory cytokines [17,23]. Such results prompted the establishment of further investigations into how the different constituents of the microenvironment may affect the proangiogenic capabilities of hiPSC-VSMC so to pave the way to the optimization of the hiPSC-based vasculature.

ECM such as collagen and fibronectin plays an active and profound role in the viability and functionalities of the stem cells, including hiPSC-VSMC [24]. We have shown that changing the environment of the collagen hydrogels, whether it is to increase the density, or implement the addition of crosslinkers, or addition of naturally derived polymers, has promoted hiPSC-VSMC’s viability and proangiogenic functions via paracrine secretions [17,25,26,27,28]. In addition to collagen, fibronectin has shown profound effects on cellular adhesion, growth, and migration, and proangiogenic function [29,30]. In a recent study, our group has demonstrated the role of fibronectin in the precise modulation of hiPSC-VSMC’s growth factor secretion with enhanced viability in a collagen hydrogel [31]. The study demonstrated the key role of integrin signaling in the precise modulation of basic fibroblast growth factor (bFGF) secretion in hiPSC-VSMC and the potential of the conditioned medium in forming EC networks in a matrigel assay. However, further studies are needed to understand the contributions of integrin signaling and hiPSC-VSMC in EC network formation.

Engineering improved pre-vascularized constructs with the support of a primed hiPSC-VSMC in a deliverable ECM-based hydrogel offer unmet opportunities to treat non-healing wounds. In the current study, we intended to investigate the effects of proangiogenic hiPSC-VSMC on EC morphogenesis. We hypothesized that proangiogenic hiPSC-VSMC provides essential cues for the growth and maintenance of EC-based networks in a fibronectin functionalized collagen hydrogel. This study is a critical step toward the fabrication of pre-vascularized tissues. Our future goal is to engineer a pre-vascularized hydrogel containing the co-culture of hiPSC-derived VSMC and EC as a viable cellular therapy to promote wound healing.

## 2. Methods

### 2.1. Cell Culture

Human iPSC-VSMC were differentiated using a previously established protocol [21]. hiPSCs were cultured in feeder-free conditions for 4 days, and then cellular colonies were collected after treating with Dispase for 15 min at 37 °C. The released cellular colonies were transferred to a low attachment plate to form embryoid bodies (EB) and treated in an mTESR medium. After 24 h, the culturing medium was changed to a mixture of mTESR and a customized EB differentiation medium (DMEM high glucose + 10% FBS + 1% non-essential amino acid (*v*/*v*) + 2 mM L-glutamine and 0.012 mM 2-mercaptoethanol) at a 1 to 3 ratio. After 72 h, the medium was changed to the customized EB differentiation medium only. The EBs were cultured for another 48 h and transferred to a gelatin-coated plated and were further cultured for another 5 days followed by culturing them on a Matrigel-coated plate for a week. The resulting differentiated cells were cultured in a commercially available smooth muscle cell medium (SmGM-2) and verified with immunofluorescence staining of SM-22α and calponin. After verification, hiPSC-VSMCs were either stored in liquid nitrogen or plated onto gelatin-coated Petri dishes.

### 2.2. HUVEC Culture

Primary human umbilical vein endothelial cells (HUVECs) were purchased from Yale Vascular Biology and Therapeutics Program. HUVECs were cultured in EC Growth Medium (EGM)-2, which was replenished every 48 h, until at least 80% confluence. All HUVECs used in our experiments were below passage number 7.

### 2.3. Fibronectin Collagen Hydrogel Production

The fibronectin functionalized collagen hydrogels (collagen + fibronectin) were produced in the following steps. The initial concentration of rat tail type-1 collagen used was 5 mg/mL. To maintain the integrity of the materials, the entire hydrogel formation process was done on ice. To achieve an overall density of 4 mg/mL collagen, the following ingredients were mixed within an Eppendorf tube in the following order: 400 µL of type-I collagen, 50 µL of MEM, then 8.4 µL of 1 M NaOH and 50 µL of 1x PBS or 1 mg/mL of fibronectin. The formulations of each hydrogel group are listed in Table 1. The mixture was gently mixed to prevent bubble formations. After the mixture color changed from yellow to light pink, respective cells, HUVEC, or a combination of hiPSC-VSMC and HUVEC of 1:4 ratio were added. The color change was due to a change in pH of the collagen solution from acidic to neutral after the addition of NaOH. HUVEC only hydrogels contained about 1 × 10^6^ cells, and combination hydrogels contained 2 × 10^5^ hiPSC-VSMC and 8 × 10^5^ HUVEC. The cell-hydrogel mixture was then further gently mixed and distributed as 100 µL hydrogel aliquots into 96 well cell culture plates containing approximately 2 × 10^5^ of total cells. The plates were then incubated in the cell culture incubator for at least 30 min at 37 °C for the gelation process. The following table contained the appropriate amount of components for each hydrogel mixture. After the hydrogels achieved gelation, 200 µL of EGM-2 or combination media (1:1 of EGM-s and SmGM-2) was added on top of the hydrogels and was incubated for 7 days, while changing media every 72 h. The various groups were HUVECs, co-culture of HUVECs and hiPSC-VSMCs in collagen hydrogels (Col-I), and collagen+fibronectin hydrogels (Col-I + Fib).

### 2.4. αvβ3 Integrin Signaling Inhibition Experiment

Another set of fibronectin functionalized collagen hydrogels containing either HUVECs or a combination of hiPSC-VSMCs and HUVECs were constructed under the same conditions as described above. 24 h after the initial culture, 2 µL echistatin (100 nM), an inhibitor of αvβ3, was added into the culturing medium and was refreshed every 2 days. At the end of the 7 days, all the hydrogels were harvested and fixed with 4% paraformaldehyde for immunofluorescence staining.

### 2.5. Immunofluorescence Staining

After day 7, the hydrogels of size 5 mm × 5 mm were harvested and washed with 1xPBS and then fixed with 4% paraformaldehyde for at least 10 min. Then the hydrogels were washed 3 times with 1xPBS for 10 min and blocked with 5% Bovine Serum Albumin in PBS and 0.05% Tween20 (BSA-PBST) for 1 h at room temperature. The hydrogels were incubated with primary antibodies of EC markers CD144, and CD31, and smooth muscle cell markers smooth muscle myosin heavy chain (SM-MHC) and SM-22-α at 1:200 dilution overnight at 4 °C. On the next day, the hydrogels were washed three times with PBST (Tween20 0.05%), and then the samples were incubated with secondary antibodies tagged with Alexa Fluor^®^ 488 for 1 h at room temperature. Dapi was used as a counterstain at 1:1000 dilution for 10 min. The hydrogels were then washed, mounted on slides, and images were taken under a confocal microscope.

### 2.6. EC Network Quantification

CD144 stained images from each hydrogel were taken from 5 different microscopic fields independently by two different lab members, and then the number of vascular networks was counted by one individual. The individual also measured and separated the sizes of the networks with a cutoff boundary at 50 µm in length. The sizes of the vascular networks (>50 µm and <50 µm) were determined by measuring the shortest side of each vascular network.

### 2.7. AlamarBlue Assay

Cell viability was performed using AlamarBlue assay following a standard method [17,28,32,33,34,35,36]. hiPSC-VSMC and HUVEC co-culture and HUVECs only in collagen and fibronectin functionalized collagen hydrogels were characterized for viability on day 7 of hydrogel culture as described [31]. First, 200 µL of culturing media was gently aspirated out for future ELISA assays. The hydrogels were then gently washed with 200 µL of 1× PBS. AlamarBlue working solution was made at a 1:10 ratio of AlamarBlue to SmGM-2 medium. 100 µL of the AlamarBlue working solution was added to each of the hydrogels and incubated at 37 °C for 2 h. After the incubation, the plate was read for fluorescence at 540 nm excitation and 590 nm emission. Relative cell viability was evaluated by normalizing measurements with the control HUVECs in collagen hydrogel fluorescence value.

### 2.8. ELISA

The conditioned medium (CM) from the hydrogels was characterized for paracrine factors using ELISA and following a standard method [17,28,37,38,39,40]. Day 3 and 7 CM collected from the culture were used to perform ELISA to assess for proangiogenic growth factors vascular endothelial growth factor (VEGF) and bFGF, as previously described [17,28]. 90 µL of the CM was placed to microwells of 96 well ELISA plates as duplicates. The plates were incubated at 4 °C overnight. The next day, the plates were washed with PBST (PBS + 0.05% Tween-20) three times and blocked using 5% BSA for 1 h at room temperature. Then the plates were washed with PBST three times and were incubated with primary antibodies (1:2500) at 4 °C overnight. The next day, the plates were washed three times with PBST and incubated with secondary antibodies conjugated with horseradish peroxidase (1:2500) for 2 h at room temperature, avoiding direct lighting whenever possible. The plates were then washed 3 times with PBST. After aspirating out the PBST, 100 µL of TMB substrate solution was added to the plates and was allowed to incubate for 25 min at room temperature on a plate shaker. 100 µL of stop solution (2N HCl) was then added to each microwell, and the absorbance was measured at 450 nm on a plate reader. The information related to primary and secondary antibodies can be found in Supplementary Tables S1 and S2. The relative level of growth factor was evaluated by normalizing with the control HUVEC collagen hydrogel absorbance value.

## 3. Results

### 3.1. Fibronectin Increased the Number of EC Networks

**Figure 1:**
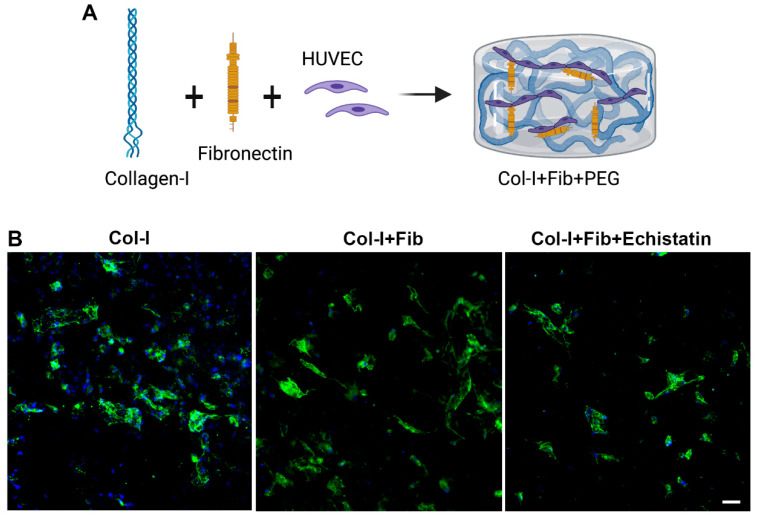
Characterization of HUVEC only hydrogels. (**A**) The schematic shows HUVEC in fibronectin functionalized collagen hydrogel-forming EC networks. (**B**) Fluorescent images of HUVEC-only hydrogels stained with CD144 on Day 7. The different hydrogels were collagen only (Col-I), fibronectin functionalized collagen hydrogel (Col-I + Fib), and Col-I + Fib hydrogel treated with Echistatin (Col-I + Fib + Echistatin). Dapi (Blue) was used to stain nuclei. The scale bar measures 50 µm.

**Figure 2:**
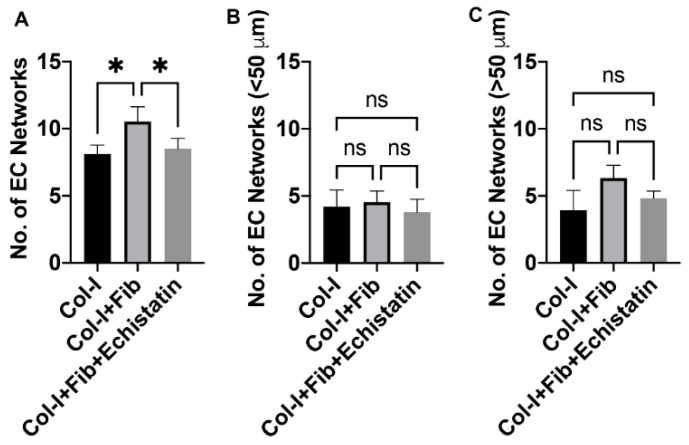
Quantification of EC networks in HUVEC only hydrogel. The graphs show quantification of (**A**) total number of EC networks, (**B**) networks less than 50 µm, and (**C**) networks more than 50 µm. The different hydrogels were collagen only (Col-I), fibronectin functionalized collagen hydrogel (Col-I + Fib), and Col-I + Fib hydrogel treated with Echistatin (Col-I + Fib + Echistatin). Statistical significance was determined using One-way ANOVA (* *p* < 0.05; *n* = 3).

The CD144 staining of the HUVEC only hydrogels demonstrates the formation of EC networks over seven days (Figure 1 and Figure 2). The fibronectin functionalized group shows an enhanced number of EC networks than the control collagen hydrogels (Figure 1B and Figure 2A). We see a modest increase in the number of networks from an average of 8 to 11. However, we do not see any difference between the number of different sizes (>50 µm and <50 µm) in both collagen only and fibronectin functionalized hydrogels (Figure 2B,C). Treatment with αvβ3 inhibitor reduced the number of EC networks in the fibronectin functionalized groups close to that of the control collagen hydrogels (B and Figure 2A). However, we do not observe a change in the size of EC networks (Figure 2B,C).

### 3.2. hiPSC-VSMC in the Presence of Fibronectin Enhanced the Number and Size of EC Networks

**Figure 3:**
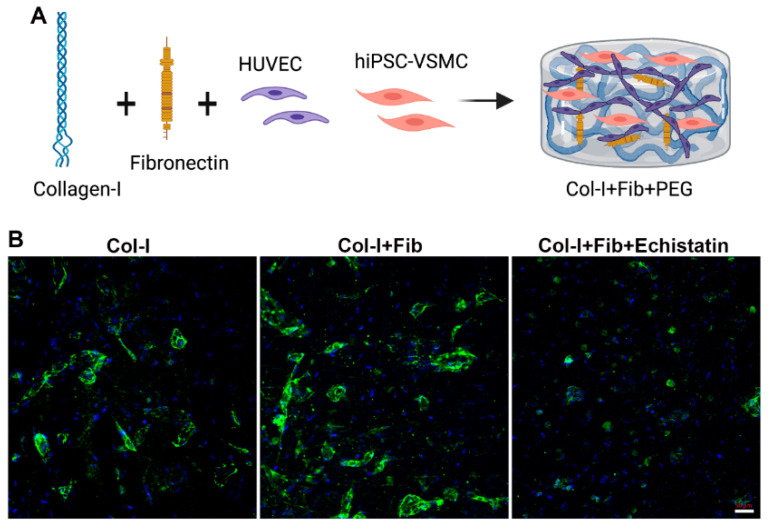
Characterization of hiPSC-VSMC and HUVEC hydrogels. (**A**) The schematic shows hiPSC-VSMC and HUVEC in fibronectin functionalized collagen hydrogel-forming EC networks. (**B**) Fluorescent images of hiPSC-VSMC and HUVEC hydrogels stained with CD144 on Day 7. The different hydrogels were collagen only (Col-I), fibronectin functionalized collagen hydrogel (Col-I + Fib), and Col-I + Fib hydrogel treated with Echistatin (Col-I + Fib + Echistatin). Dapi (Blue) was used to stain nuclei. The scale bar measures 50 µm.

**Figure 4:**
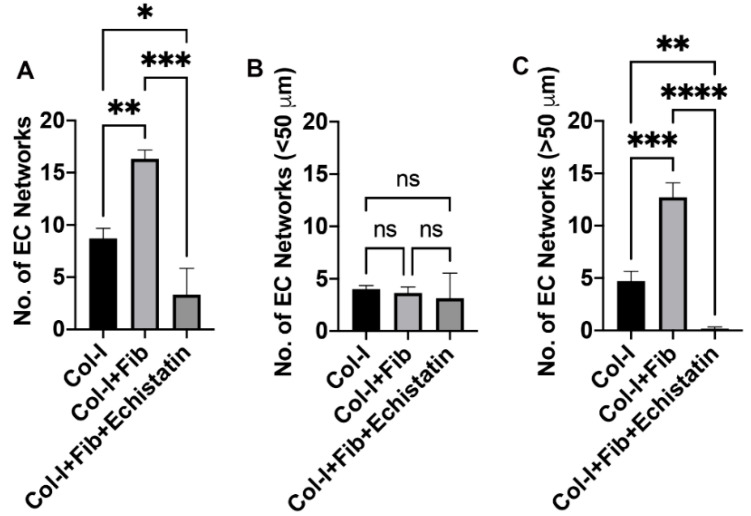
Quantification of EC networks in hiPSC-VSMC and HUVEC hydrogels. The graphs show quantification of (**A**) total number of EC networks, (**B**) networks less than 50 µm, and (**C**) networks more than 50 µm. The different hydrogels were collagen only (Col-I), fibronectin functionalized collagen hydrogel (Col-I + Fib), and Col-I + Fib hydrogel treated with Echistatin (Col-I + Fib + Echistatin). Statistical significance was determined using One-way ANOVA (* *p* < 0.05; ** *p* < 0.01; *** *p* < 0.001; **** *p* < 0.0001; *n* = 3).

We quantified the number of CD144 stained EC networks in the co-culture of hiPSC-VSMC and HUVEC in the fibronectin functionalized and control hydrogels (Figure 3 and Figure 4). We see a similar trend as HUVEC-only culture in the fibronectin functionalized hydrogel compared to control (Figure 3B and Figure 4A). However, the co-culture of hiPSC-VSMC and HUVEC shows a substantial increase in the number of EC networks from an average of 8 to 16 (Figure 4A). We do not observe any significant difference in the number of smaller EC networks (<50 µm) between fibronectin functionalized and collagen hydrogels (Figure 4B). At the same time, we see nearly 78% of the total EC networks are larger than 50 µm in size in the fibronectin group compared to 54% in the case of control collagen hydrogels (Figure 4C). The number of >50 µm EC networks is three times that of control collagen hydrogels (Figure 4C). Treatment with αvβ3 inhibitor reduced the EC networks in the fibronectin functionalized groups by close to 20% and affected their size (Figure 3B and Figure 4A–C).

### 3.3. hiPSC-VSMC Irrespective of Fibronectin Enhanced the Viability and Proangiogenic Growth Factor Secretion

**Figure 5:**
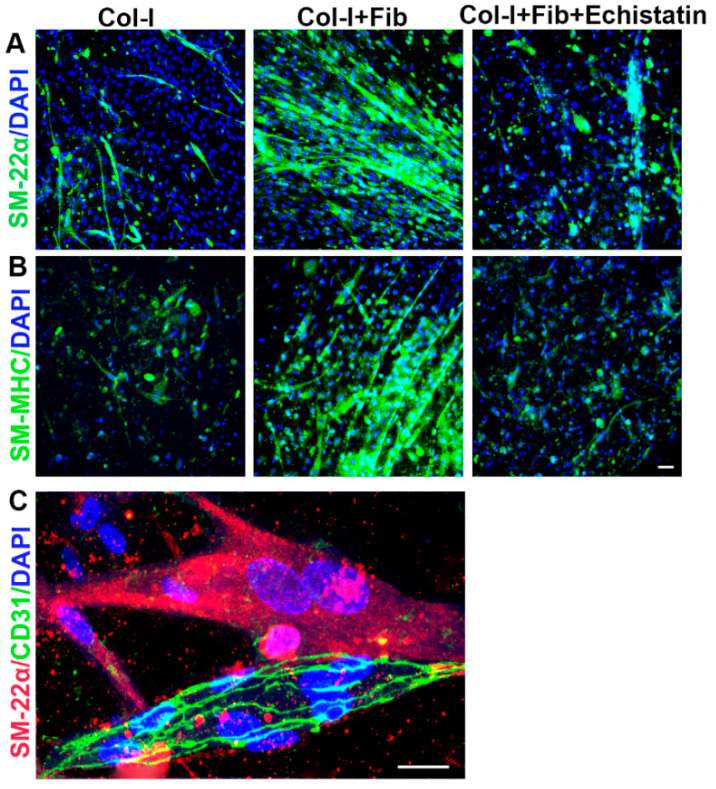
Characterization of cell phenotype embedded in the co-culture hydrogels of hiPSC-VSMC and HUVEC. Phenotype assessment of hiPSC-VSMCs embedded in the hydrogels. Immunofluorescence images showing (**A**) SM-22α (Green), and (**B**) smooth muscle myosin heavy chain (Green) stained hiPSC-VSMC in the co-culture hydrogels. The groups were collagen hydrogel (Col-I), fibronectin functionalized collagen hydrogels (Col-I + Fib), and echistatin treated Col-I + Fib hydrogels (Col-I + Fib + Echistatin). Dapi (Blue) was used to stain nuclei. Scale bar measures 50 µm. (**C**) Representative immunofluorescence images showing staining of both hiPSC-VSMC and HUVEC with SM-22α (Red) and CD31 (Green), respectively, in a Col-I + Fib hydrogel. Dapi (Blue) was used to stain nuclei. Scale bar measures 20 µm.

We stained the cells in the co-culture of hiPSC-VSMC and HUVECs with VSMC specific markers such as SM-MHC and SM-22α (Figure 5). We could identify the presence of hiPSC-VSMCs based on their staining with SM-22α (Figure 5A) and SM-MHC (Figure 5B) in collagen-only hydrogels, fibronectin functionalized collagen hydrogels, and fibronectin hydrogels treated with echistatin. Fibronectin functionalized hydrogels showed more cells compared to the control collagen and echistatin treated hydrogels in the case of both SM-22α (Figure 5A) and SM-MHC (Figure 5B). The hiPSC-VSMCs in the fibronectin hydrogels were also seen to be more aligned compared to control and echistatin-treated hydrogels (Figure 5A,B). Figure 5C shows the co-staining of hiPSC-VSMC with SM-22α and EC network with CD31 in the case of fibronectin functionalized hydrogel.

**Figure 6:**
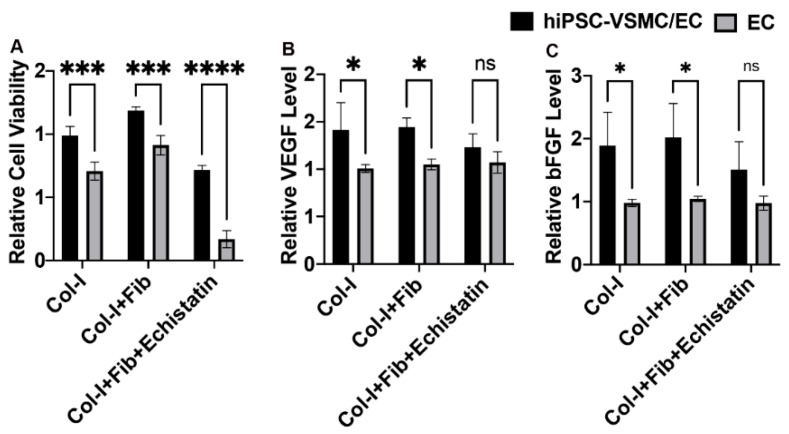
Characterization of cell viability and paracrine secretion. (**A**) The AlamarBlue assay shows the relative viability of the HUVEC and co-culture hydrogels. ELISA data showing relative secretion of relative levels of growth factors (**B**) VEGF, and (**C**) bFGF. The hydrogel groups were collagen hydrogel (Col-I), fibronectin functionalized collagen hydrogels (Col-I + Fib), and echistatin treated Col-I + Fib hydrogels (Col-I + Fib + Echistatin). * denotes statistical significance differences between the different groups (*n* = 3; one-way ANOVA, * *p* < 0.05; *** *p* < 0.001, **** *p* < 0.0001).

Relative cell viability was determined using AlamarBlue assay (Figure 6A). The co-culture of hiPSC-VSMC and HUVECS and HUVECs only cultured on collagen-only hydrogels, fibronectin functionalized collagen hydrogels, and fibronectin hydrogels treated with echistatin were tested for cell viability. The co-culture showed enhanced cell viability compared to HUVECs only in the case of control and fibronectin functionalized collagen hydrogels. The echistatin treatment reduced the cell viability for both co-culture and HUVECs only hydrogels. However, the reduction was in the case of HUVECs only. In addition, the cell viability was found to be more in the case of fibronectin functionalized hydrogels compared to control and echistatin treated hydrogels in the case of both co-culture and HUVECs only. The control hydrogels had more cell viability than the echistatin-treated hydrogels.

VEGF and bFGF growth factor level in the CM of the co-culture of hiPSC-VSMC and HUVECS and HUVECs only cultured on collagen only hydrogels, fibronectin functionalized collagen hydrogels, and fibronectin hydrogels treated with echistatin were characterized (Figure 6B,C). VEGF was shown to be secreted at an increased level in the case of co-culture compared to HUVECs only in both fibronectin and control collagen hydrogels (Figure 6B). A similar trend can be found in the case of bFGF (Figure 6C). However, echistatin treatment reduced the level of both VEGF and bFGF secretion (Figure 6B,C). There was also no difference found between control and fibronectin collagen hydrogels in the level of VEGF and bFGF secretion. The level of VEGF and bFGF secretion of HUVECs only hydrogel was unaffected by the culture conditions and was at a similar level among control, fibronectin functionalized, and echistatin treated hydrogels (Figure 6B,C).

## 4. Discussion

This study aims at optimizing a proangiogenic hydrogel using fibronectin and hiPSC-VSMC. Human iPSC-VSMC is currently an attractive candidate for pathophysiological disease modeling and potentially a factor for vascular regenerative therapy [25]. We have developed a robust protocol to generate a pure population of hiPSC-VSMCs without the need for additional purifications steps. These hiPSC-VSMCs have already been used in developing disease models, vascular grafts, and therapies for wound healing. Several studies have investigated how various properties of ECM may dictate cellular functions, thus drawing the importance of ECM to cell relationships [26,27,28,29]. To realize the VSMC’s full potential, there is a need to further optimize the ECM environment in which the VSMC can survive and secrete appropriate proangiogenic paracrine factors. In previous studies, we have demonstrated that increasing the density of the collagen 3D hydrogels resulted in augmenting VSMC’s secretion of several proangiogenic growth factors, including VEGF, bFGF, ANG-1, PDGF, TGF-β1, SDF-1α, and MMP-2 [11]. Fibronectin, an ECM protein, regulates the proangiogenic function of EC and maintains their fenestrae structures [7,30]. We recently showed that fibronectin modulates VSMC function via αvβ3 [31] and promotes viability and bFGF secretion. Fibronectin with both integrin and growth factor-binding motifs in its protein sequence is thus an attractive biomolecule to engineer a microenvironment to develop pre-vascularized hydrogels containing hiPSC-VSMC.

In our study, we characterized the viability, growth factor secretion, and network formation of HUVECs in the presence of hiPSC-VSMC within a fibronectin functionalized 3D collagen environment. We demonstrated that with the lack of fibronectin contacts via the inhibitory factor echistatin, there was a significant decrease in the overall viability of HUVEC. Echistatin is an inhibitor of αvβ3 integrin, at which fibronectin may potentially interact with HUVEC to support the cells’ survivability. It is known that HUVEC themselves inherently secrete a basal amount of fibronectin [32]. This would explain why the HUVEC control group exhibited higher levels of cellular viability than the echistatin group. Our data further suggested that the presence of interactable fibronectin is essential for the survivability of HUVEC. This indicated that fibronectin and interaction with αvβ3 integrin are required for EC to be self-sufficient in maintaining their viability. We also demonstrated that in a fibronectin functionalized 3D environment, HUVEC was able to form more EC network aggregates. However, we did not see any effect of fibronectin on the secretion of proangiogenic paracrine factors such as VEGF and bFGF. We thus hypothesized that an increased network formation might be due to fibronectin’s inherent pro-adhesive properties and not secretory factors. The fibronectin functionalized matrix might be providing the necessary foundation to stabilize HUVEC allowing adjacent cells to more readily connect and form EC network aggregates. Overall, fibronectin interaction with HUVEC via αvβ3 integrin is essential for the cells’ viability, and fibronectin provides a more suitable environment for HUVEC formation of vascular networks.

Several previous studies demonstrated the structural supportive behaviors of VSMC to ECs [33,34]. The next avenue to look at was how ECs respond to VSMC’s presence and its paracrine secretions. We recently demonstrated that fibronectin primes hiPSC-VSMC for enhanced paracrine secretion. We attempted to use hiPSC-VSMCs in the presence of fibronectin to regulate EC morphogenesis. The hiPSC-VSMC irrespective of fibronectin, significantly improved the viability of the EC in the co-culture. Similar to HUVEC-only fibronectin functionalized hydrogels, the co-culture hydrogels demonstrated a significant increase in the number of EC vascular network formations. In addition to the total increase in EC networks, the larger size of EC networks was also significantly more in the fibronectin functionalized hydrogels compared to control. Interestingly, the number of larger EC networks in echistatin hydrogels dropped down to almost nonexistent. This observation is a stark departure from the HUVEC-only echistatin hydrogels. We believe that in the presence of hiPSC-VSMC an increase in the total vascular networks was driven by the significant increase in VEGF and bFGF secretion. With the addition of echistatin, an inhibitor of αvβ3 integrin, there was a significant decrease in the total number of vascular networks, which is driven by the total elimination of large vascular networks. These data indicate the lack of VEGF and bFGF stimulation from hiPSC-VSMC may also contribute to the HUVEC’s dysfunction in the maintenance of larger vascular networks. In an older study, the formation of such capillary-like cord structures was synergistically stimulated by VEGF and bFGF [35]. This further shows the adverse effects of an environment devoid of interactable fibronectin on hiPSC-VSMC and EC, decreasing their viability and paracrine capabilities. Our previous study also has shown decreased cellular viability and bFGF secretion in the presence of echistatin [36]. Combining previous results and our present data, the overarching idea may be how vital fibronectin and hiPSC-VSMC are in the viability and formation of EC networks.

Our present study further indicated that the decrease in VSMC’s capabilities is due to the lack of interactable fibronectin that actively suppressed the functions of HUVEC nearby, resulting in obliteration of larger cord network formation. However, smaller cord network formations were not negatively affected by the inhibitory effect of echistatin in both the HUVEC-only and combination cell fibronectin functionalized hydrogels. This may be due to the inherent ability of HUVEC to self-accumulate via their residual adhesive surface markers such as intercellular adhesion molecule-1 (ICAM-1), vascular cell adhesion molecule-1 (VCAM-1), E-selectin, and P-selectin [37]. From our studies, any further expansion of the initial vascular networks may require the aid of fibronectin interaction and the functionality of viable vascular smooth muscle cells.

## 5. Conclusions

Collectively, our data indicate that hiPSC-VSMC in the presence of fibronectin actively enhances the morphogenesis of HUVEC in forming large vascular networks. Our study further suggests the importance of fibronectin and hiPSC-VSMC interaction and paracrine secretion in EC morphogenesis. This finding is a first and critical step towards developing an improved pre-vascularized hydrogel for wound healing. Future studies involve using hiPSC-derived Ecs for the development of an autologous source of the pre-vascularized hydrogel.

## Figures and Tables

**Table 1:** Various formulations of collagen + fibronectin hydrogels with hiPSC-VSMCs and HUVECs in a total volume of 500 µL.

Samples	Density (mg/mL)	Collagen (µL)	10× MEM (µL)	10× PBS (µL)	1 M NaOH (µL)	Fibronectin 1 mg/mL (µL)	hiPSC VSMC 8 × 10^3^/µL	HUVEC 16 × 10^3^/µL
Col-I+EC	4	400	50	50	8.4	0	0	62.5
Col-I+Fib+EC	4	400	50	0	8.9	50	0	62.5
Col-I+iPSC-VSMC+EC	4	400	50	50	8.4	0	25	50
Col-I+Fib+iPSC-VSMC+EC	4	400	50	0	8.9	50	25	50

## Data Availability

The data presented in this study are available in the manuscript and Supplementary Information. Additonal information can be obtained upon request from the corresponding authors.

## Supplementary Materials

The following are available online at https://www.mdpi.com/article/10.3390/bioengineering8120223/s1, Table S1: Information related to primary antibodies, Table S2: Information related to secondary antibodies.
