# Supplementary material for: Human iPSC-Derived Vascular Smooth Muscle Cells in a Fibronectin Functionalized Collagen Hydrogel Augment Endothelial Cell Morphogenesis
Source: Bioengineering (Basel). 2021 Dec 18;8(12):223. doi: 10.3390/bioengineering8120223 (PMC8698933; doi:10.3390/bioengineering8120223)
Supplement: Supplementary file 1 [file bioengineering-08-00223-s001.zip › bioengineering-1491746-supplementary.pdf]

bioengineering

Article

Human iPSC-Derived Vascular Smooth Muscle Cells in a Fibronectin Functionalized Collagen Hydrogel Augment Endothelial Cell Morphogenesis

Kaiti Duan [1,†], Biraja C. Dash [1,*,†], Daniel C. Sasson [1], Sara Islam [1], Jackson Parker [1] and Henry C. Hsia [1,2,*]

[1] Section of Plastic Surgery, Department of Surgery Yale School of Medicine, Yale University, New Haven, CT 06510, USA; kaiti.duan@yale.edu (K.D.); dcsasson@gmail.com (D.C.S.); sara.islam@yale.edu (S.I.); jackson.parker@yale.edu (J.P.)
[2] Department of Biomedical Engineering, Yale University, New Haven, CT 06511, USA
* Correspondence: biraja.dash@yale.edu (B.C.D.); henry.hsia@yale.edu (H.C.H.); Tel.: +1-203-737-2049 (H.C.H.)
† Equal Contribution.

Running Title: hiPSC-VSMC Promotes EC Morphogenesis

Supplementary Materials:

Vascular smooth muscle cell (SmGM-2) and endothelial cell (EGM-2) growth media are purchased from Promocell, Germany. Type-I collagen from Enzo Life Sciences, USA; mTESR medium and dispase from STEMCELL Technologies; human plasma fibronectin from Millipore Sigma; Echistatin from R&D Systems; Ultra-low attachment and ELISA (NUNC) plates from Thermo Fisher Scientific; matrigel from Corning; TMB substrate from Abcam; and AlamarBlue from Thermo Fisher Scientific. All other reagents and laboratory supplies were purchased from Thermo Fisher Scientific, USA.

Table S1. Information related to primary antibodies.

Primary Antibody	Dilution	Catalog Number/ Manufacturer
bFGF	1:2500 ELISA	500M38 (PeproTech)
VEGF	1:2500 ELISA	AB-119 (Abcam)
SM-22α	1:200 IF	AB-10135 (Abcam)
CD31	1:200 IF	sc-376764 (Santacruz)
CD144	1:200 IF	sc-9989 (Santacruz)

Table S2. Information related to secondary antibodies.

Secondary Antibody	Dilution	Catalog Number/ Manufacturer
Anti-Mouse-HRP	1:2500 ELISA	AB6789 (Abcam)
Anti-Mouse-Alexafluor488	1:250 IF	A21202 (ThermoFisher)
Anti-Goat-Alexafluor555	1:250 IF	A21432 (ThermoFisher)
